# Electrochemical Behavior of Quinoxalin-2-one Derivatives at Mercury Electrodes and Its Analytical Use

**DOI:** 10.1100/2012/409378

**Published:** 2012-04-30

**Authors:** Milan Zimpl, Jana Skopalova, David Jirovsky, Petr Bartak, Tomas Navratil, Jana Sedonikova, Milan Kotoucek

**Affiliations:** ^1^Department of Analytical Chemistry, Faculty of Science, Palacky University in Olomouc, 17. listopadu 12, 771 46 Olomouc, Czech Republic; ^2^Regional Centre of Advanced Technologies and Materials, Department of Analytical Chemistry, Faculty of Science, Palacky University, 17. listopadu 12, 771 46 Olomouc, Czech Republic; ^3^J. Heyrovsky Institute of Physical Chemistry of the ASCR, v. v. i., Dolejskova 3, 182 23 Prague 8, Czech Republic

## Abstract

Derivatives of quinoxalin-2-one are interesting compounds with potential pharmacological activity. From this point of view, understanding of their electrochemical behavior is of great importance. In the present paper, a mechanism of electrochemical reduction of quinoxalin-2-one derivatives at mercury dropping electrode was proposed. Pyrazine ring was found to be the main electroactive center undergoing a pH-dependent two-electron reduction process. The molecule protonization of nitrogen in the position 4 precedes the electron acceptance forming a semiquinone radical intermediate which is relatively stable in acidic solutions. Its further reduction is manifested by separated current signal. A positive mesomeric effect of the nonprotonized amino group in the position 7 of the derivative III accelerates the semiquinone reduction yielding a single current wave. The suggested reaction mechanism was verified by means of direct current polarography, differential pulse, cyclic and elimination voltammetry, and coulometry with subsequent GC/MS analysis. The understanding of the mechanism was applied in developing of analytical method for the determination of the studied compounds.

## 1. Introduction

The identification and determination of trace amounts of biologically active substances and their metabolites represent one of the most important problems of contemporary analytical chemistry. Extra attention is paid to the compounds exhibiting bacteriostatic, virostatic, and cancerostatic effects. These include compounds capable of interactions (via intermolecular hydrogen bonds) with binding sites of nucleic acids intervening in their primary functions [1, 2]. This phenomenon can be used in targeted cancer therapy, especially if tumor cells exhibit resistance towards conventional chemotherapeutic treatment.

The parent quinoxaline-2-one has become one of the active components of the preparation whose activity against HIV-virus was tested [3]. Natural quinoxaline analogues, such as quinomycin antibiotics (equinomycin) and triostines, belong among well-known compounds with confirmed virostatic and bacteriostatic activity [4–6]. Similar effects can also be expected for synthetic 3 substituted quinoxaline-2-ones. Some of their analogues are used (with certain limitations) in agriculture and veterinary medicine, others as fluorescent derivative agents in HPLC [7].

Much of the complex quinoxaline metabolism remains to be understood. A partial approximation of the biotransformation mechanism can be achieved using the electrochemical charge-transfer model in which the electrode can be considered a simplified biological receptor.

**Figure 1:**
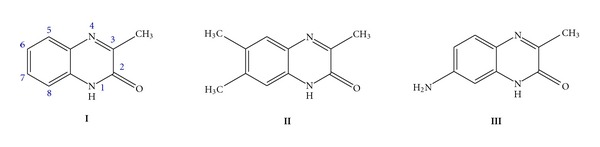
Chemical structures of 3-methylquinoxalin-2(1*H*)-one (**I**), 3,6,7-trimethylquinoxalin-2(1*H*)-one (**II**), and a 7-amino-3-methylquinoxalin-2(1*H*)-one (**III**).

Few references can be found regarding the electrochemical behavior of 3 substituted quinoxaline derivatives [8, 9]. Two-electron irreversible polarographic reduction of 3-methylquinoxalin-2(1*H*)-one has been reported to proceed in the pH range of 0–14. A radical intermediate forming in the first step of the electroreduction in strongly acidic media can either receive an electron and a proton in the second step or form a N,N′-dimer dependently on quinoxalinone concentration [8, 9]. The aim of this paper is to contribute to the explanation of electrochemical behavior of quinoxaline-2-one derivatives (**I–III**, Figure 1) at the mercury electrode, and to identify both the intermediate and final products of their electrochemical reduction.

## 2. Experimental

### 2.1. Instrumentation

A Radelkis OH 102 polarograph (Radelkis, Budapest) in a three-electrode arrangement with dropping mercury electrode (DME, *t* = 3.6 s, *m* = 1.98 mg/s), auxiliary platinum and reference-saturated calomel electrode (SCE) was used for the recording of DC curves.

An Eco-Tribo Polarograph with pencil mercury microelectrode (Polaro-Sensors, Prague) was used for the DC-tast polarography, differential pulse (DP) voltammetry (potential scan rate 20 mV/s, modulation amplitude –50 mV), adsorptive stripping (AdSV) and cyclic voltammetry (CV) measurements. The same reference and auxiliary electrodes were used as those described above.

The computation of data filtering and elimination equations was performed using self-made macro in Microsoft Office Excel v. 2003 (Microsoft Corporation, USA).

Controlled potential coulometry measurements were carried out using an OH 404 analyzer (Radelkis, Budapest) equipped with a mercury pool cathode (26.4 cm^2^). The SCE was used as a reference. The platinum auxiliary electrode was placed in the anodic compartment separated with a frit.

Before the analysis, all samples were deaerated with a nitrogen stream for 10 min.

For GC/MS identification of coulometric reduction products, an HP 6890 chromatograph with an HP 7683 injector and an Agilent 5973 N mass spectrometer (all Agilent, Palo Alto, USA) were used. Chromatographic conditions: HP-5 MS column (30 m × 0.25 mm × 0.25 *μ*m), helium as a carrier gas (99.998%, SIAD, Bergamo, Italy), flow-rate 0.9 mL/min. Programmed temperature was 50°C/2 min–10°C/min up to 300°C and 5 min isothermically. 

Spectrophotometric data were acquired by a Philips PU 8755 UV/VIS spectrophotometer (Cambridge, UK) and a Beckman DU 7500 spectrophotometer (Fullerton, USA), using quartz cuvettes.

A calibrated inoLab pH Level 1 equipped with a SenTix 41 combined electrode (WTW, Weilheim, Germany) was used for pH measurements.

### 2.2. Chemicals and Reagents

Standards of quinoxaline-2(1*H*)-one derivatives were synthesized and purified at the Department of Organic Chemistry, Palacky University, Olomouc. The purity (>97%) of the standards obtained was verified by checking the melting points, IR spectrometry (KBr technique) and by HPLC-MS.

Stock methanolic solutions (1 mM) were further diluted by methanol if needed. In cases when analyte concentrations exceeded 1 *μ*M, a methanol addition (10%, v/v) was necessary to maintain homogeneous solution because of the low water solubility of the studied compounds.

Britton-Robinson buffer solutions with ion strength corrected by KCl were used for pH adjustments of the samples. For measurements at strong acidic or alkaline conditions, diluted solutions of HCl or NaOH were used. Ultra pure water (0.055 *μ*S/cm) from ELGA system was used for the solution preparation.

### 2.3. Procedures

Coulometric experiments were performed with three different electrolytes and different constant potentials: 0.01 M HCl (*E* = −0.8  *V*), 0.001 M NaOH (*E* = −1.6  *V*), phosphate buffer, and pH 7.0 (*E* = −1.2  *V*). During electrolysis (in the interval from 0 to 60 min), 10 mL samples were withdrawn, passed through a preconditioned Zorbax C18 SPE cartridge (200 mg, Agilent, Palo Alto, USA) and dried with a vacuum water pump. Retained compounds were eluted using 1 mL of methanol and directly analyzed by GC/MS.

Liquid-liquid extraction of quinoxalinones from spiked water samples (8–20 *μ*g of I per 100 mL water) was performed using a 250 mL separatory funnel. A volume of 100 mL of ethylacetate was added to 50 mL of spiked water sample in the funnel and shaken for 5 min. The upper analyte-containing organic layer was removed, transferred into a dryer and evaporated to dryness under reduced pressure at 70°C. The sample was then reconstituted in 1 mL of methanol, diluted in 10 mL of the background electrolyte (1 mM NaOH) and analyzed using DPV. The standard addition method was used for the quantification.

### 2.4. Results and Discussion

#### 2.4.1. Mechanism of Electrode Reaction

**Figure 2:**
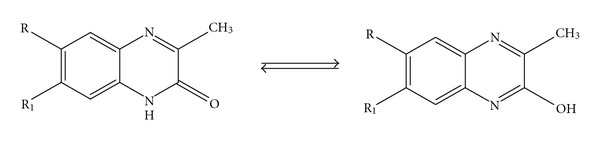
Keto-enol-tautomery of quinoxalin-2(1*H*)-one derivatives.

Electron deficiency in a molecule is essential for its electrochemical reduction. For quinoxaline derivatives, the electron deficiency is set by their quinoid structure. An influence of keto-enol-tautomery should be considered for the compounds of interest (Figure 2).

Although some earlier studies reported these compounds also as hydroxo derivatives [10], many properties (reactivity, IR-spectra) indicate that the keto form prevails [11, 12]. Hydrogen bond is likely to play a significant role in the reduction process, stabilizing the planar molecule.

**Figure 3:**
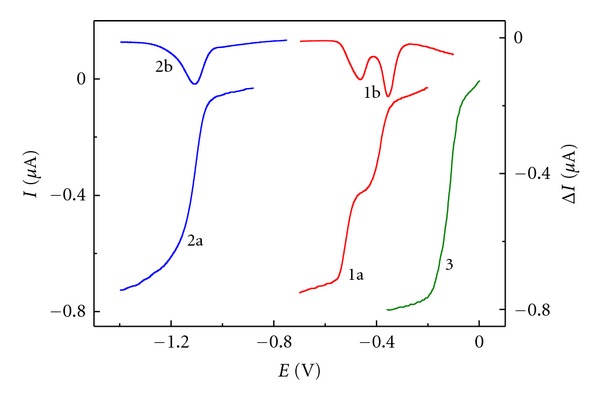
DC-tast polarographic curves of 3,6,7-trimethylquinoxalin-2(1*H*)-one (**II**) in 0.5 M HCl (1a), in Britton-Robinson buffer pH 11.5 (2a) and benzil curve of in 0.5 M HCl (3).

Quinoxalin-2(1*H*)-one derivatives are reduced within the whole acidic range. In acidic solutions, diffusion-controlled electrode process is manifested by a dual wave (Figure 3, curves (1a) and (1b). Both waves are approaching each other as acidity decreases, whereas in the alkaline range (for **III** in neutral) they merge into one single DC-wave or DPV-peak (Figure 3, curves (2a) and (2b), resp.). With increasing pH, a potential shift towards more negative values occurs, indicating that protons are involved in the electrode process.

DP voltammetric curves of **II** in 0.5 M HCl (1b) and in Britton-Robinson buffer pH 11.5 (2b).

Depolarizer concentration is 0.1 mM, methanol content 10% (v/v).

**Figure 4:**
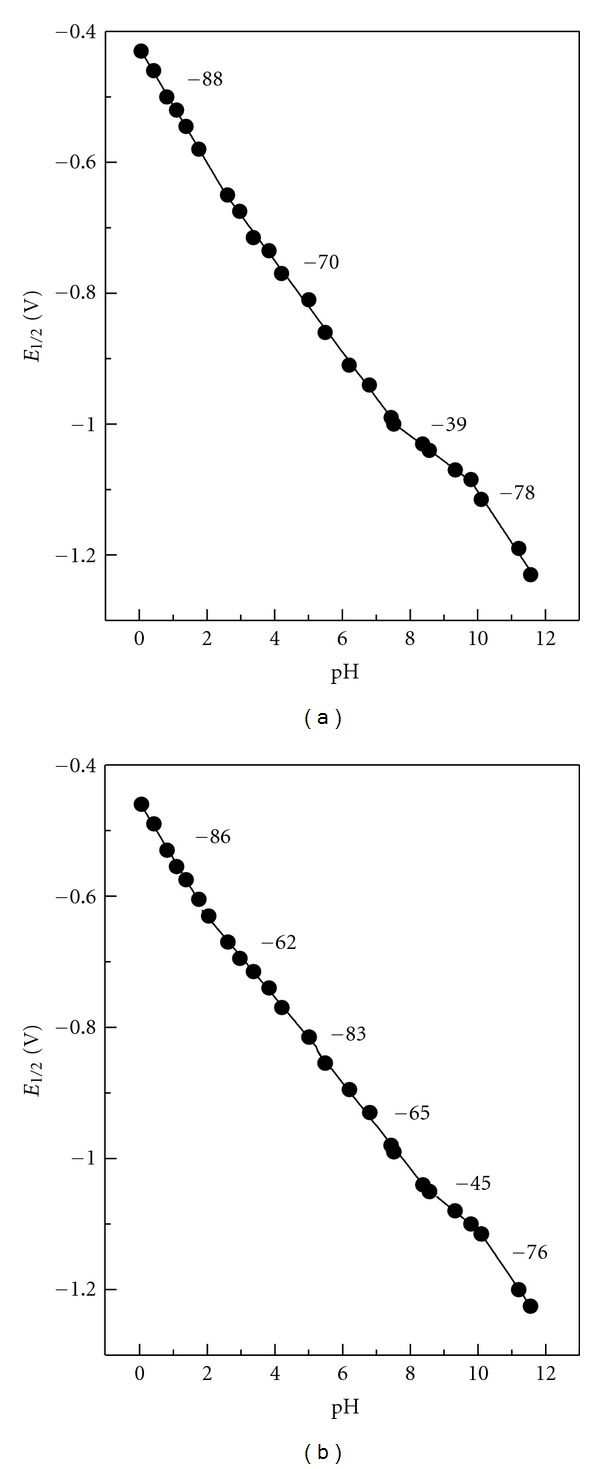
Dependencies of half-wave potentials of DC-waves for **I** (a) and **III** (b) on pH in HCl and Britton-Robinson solutions. The numbers indicate slopes (mV/pH) of corresponding linear segments.

To determine the dissociation constant by spectrophotometry, modified Henderson-Hasselbalch equation was used:

(1) $$pK_{a}=\text{pH}-\log\frac{A-A_{1}}{A_{2}-A},$$

where *A* is absorbance at given pH value, *A*_1_ and *A*_2_ are absorbances of solutions containing more than 99 % undissociated and dissociated forms, respectively. Dissociation constants of derivatives** I **(*pK*_*a*_ = 9.85) and** II** (*pK*_*a*_ = 10.04) were established in 10% (v/v) methanol. The dissociation constant of the derivative I was found to be in agreement with the reported value (*pK*_*a*_ = 9.90) [10]. The estimation of dissociation constant of the derivative **III** by spectral method was not successful. The *pK*_*a*_ values found were verified on the basis of the *E*_1∕2_ dependency of the total DC-tast wave on pH. For analytes** I **and **II**, dissociation constants both of the oxidized forms and the two dissociation constants of the reduced forms were determined by the intercepts of four linear segments of the functional dependency (Figure 4(a)).

The found dissociation constants of the oxidized forms of **I **and** II **are in good agreement with the spectrophotometric method. In case of **III**, additional protolytic equilibrium is produced as a result of the protophilic property of the amino group. Therefore, two dissociation constants of the oxidized form, and three dissociation constants of the reduced form were established from the intercepts of *E*_1/2_ = *f*(pH) dependence.

The curve break (Figure 4(b)) corresponds with *pK*_R2_. However, it is situated close to *pK*_Ox1_, and thus its position is difficult to identify with certainty due to low experimental point count. An overview of dissociation constants obtained polarographically is given in Table 1.

To evaluate independently the expected two-electron reduction of the analytes studied, the following methods were used.

**Figure 5:**
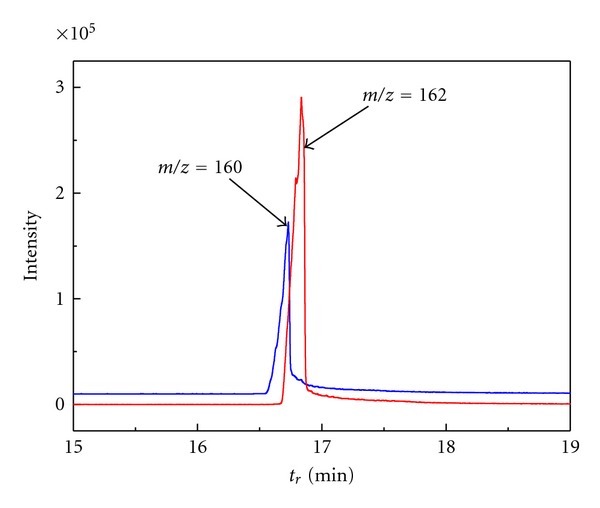
Reconstructed chromatograms for the selected *m*/*z* values. The data were obtained by GC/MS analysis of **I** (0.1 mM) electrolyzed at constant potential −0.8 V in acidic solution (0.01 M HCl in water with 10% (v/v) of methanol).

**Figure 6:**
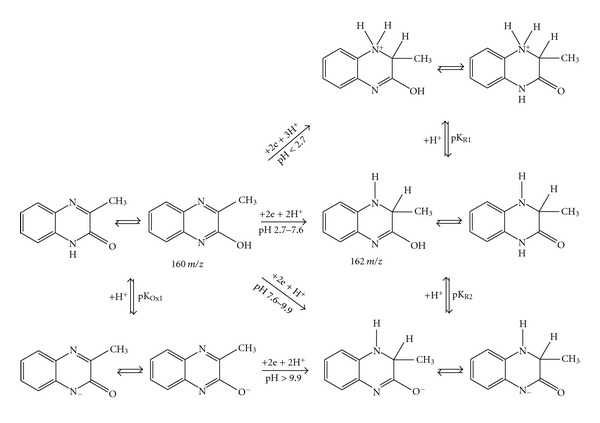
Proposed scheme of electrochemical reduction of 3-methylquinoxalin-2(1*H*)-one (**I**).

(1) A comparison of cathodic DC-tast waves of analytes with two-electron wave of benzil or with four-electron wave of *m*-nitrobenzoic acid, respectively was performed (Table 2).
(2) Potentiostatic coulometry at mercury pool cathode was performed of** I **and** II **(*c* = 0.1 mM) with subsequent GC/MS analysis. The products with *m*/*z *increased by 2 Da were found in electrolyzed solution (Figure 5) which corresponds to the two-electron reduction. They were identified as respective 3,4-dihydroquinoxalin-2(1*H*)-ones (see Figure 6).

The amino derivative **III** did not yield sufficiently volatile products, and was therefore not analyzed using this method. Nevertheless, its similar behavior at mercury electrode indicates that the two-electron reduction can also be expected. 

Based on the experimental results and considering the stabilizing effects of tautomery and mesomery, following mechanism of electrode reduction process can be concluded for the** I **and** II **derivatives (Figure 6).

**Figure 7:**
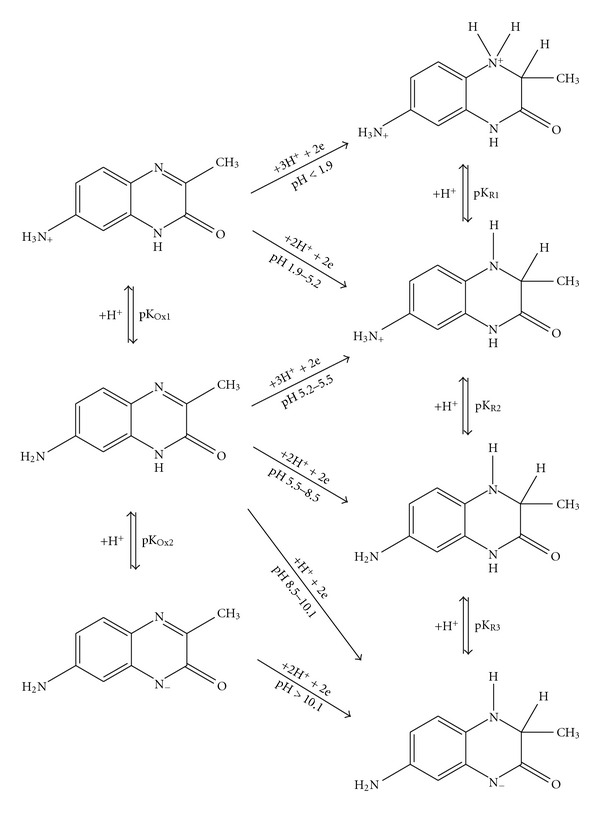
Proposed scheme of electrochemical reduction of 7-amino-3-methylquinoxalin-2(1*H*)-one (**III**).

Rather more complex mechanism can be expected for **III** where amino group is also involved in the protolytic equilibria. However, deprotonization of the  NH_3_^+^ group of the reduced form, which is probably allied to that of oxidized form, is not obvious in the *E*_1/2_(DC-tast) = *f*(pH) dependency. Based on these observations, supposed reaction scheme for **III** (excluding tautomery and mesomery) can be deduced (Figure 7). 

As the appropriate slopes of linear segments in the *E*_1/2_= *f*(pH) dependency rather differ from the theoretical values for the reversible diffusion controlled processes, it can be assumed that electrode reduction of **I–III **does not exhibit a purely reversible character. In case of irreversible reduction, *E*_1/2_  = *f*(pH) dependency is defined by (2) [13, 14]:

(2) $$E_{1/2}=E^{f}-p\frac{0.0592}{\alpha z}\text{pH},\;$$

where *p* is number of protons, *α* is a charge transfer coefficient,  *E* ^*f*^ is potential of *E*_1/2_ = *f*(pH) at *pH* = 0  as read off from the graph. The product of parameters **α*z* (Table 3) was determined from DC-curves:

(3) $$\begin{array}{cc} \;\left(\text{a}\right) & \;E_{1/4}-E_{1/2}=\frac{0.0517}{\alpha z},\;\text{or}\\ \;\left(\text{b}\right) & {\;E}_{1/2}-E=\frac{0.0592}{\alpha z}\log\frac{2x\left(3x-1\right)}{5\left(1-x\right)},\;\;\;\;x=\frac{I_{E}}{I_{\lim}\;}, \end{array}\;$$

where *I*_*E*_ is the current obtained at a potential *E* and *I*_lim⁡_ denotes the limiting current. 

Individual linear slopes of *E*_1/2_ = *f*(pH) (Figure 4) correspond to those calculated using (4) [13]:

(4) $$\frac{dE_{1/2}}{d\text{pH}}=-0.0592\frac{p}{\alpha z},$$

confirming the supposed mechanism of the overall reduction of **I–III **at mercury dropping electrode. 

In acidic media, the total two-electron reduction is divided into two one-electron steps exhibiting separate waves at low pH values (Figure 3). Considering the polarization of curve shape and taking into account the behavioral analogies of related quinoid compounds [15, 16], it is obvious that the intermediate reduction product is semiquinone radical. 

Consecutive reduction via semiquinone (Sem) can be described as follows:

(5) $$\begin{array}{c} \text{Ox}\;+\;\text{e}\;\Leftrightarrow \text{Sem},\\ \text{Sem}\;+\;\text{e}\;\Leftrightarrow \text{Red}. \end{array}$$

The equilibrium of all three forms:

(6) Ox  +  Red  ⇔  2Sem

is characterized by a semiquinone formation constant *K:*

(7) $$K=\frac{{\left[\text{Sem}\right]}^{2}}{\left[\text{Ox}\right]\left[\text{Red}\right]}.$$

Semiquinone formation can also be characterized by the dependency of formal half-wave potentials of both partial reactions (*E*_1_ and *E*_2_). Both potentials will be situated symmetrically around the mean inflection point (i.e., *≈E*_1/2_). For the equilibrium is valid [17]:

(8) $$\;\begin{array}{cc} E=E_{1}+\frac{RT}{F}\ln\frac{\left[\text{Ox}\right]}{\left[\text{Sem}\right]} & =E_{1/2}+\frac{RT}{2F}\ln\frac{\left[\text{Ox}\right]}{\left[\text{Red}\right]}\\ & =E_{2}+\frac{RT}{F}\ln\frac{\left[\text{Sem}\right]}{\left[\text{Red}\right]}, \end{array}\;$$

(9) $$E_{1}=E_{1/2}+\frac{RT}{2F}\;,$$

and similarly,

(10) $$E_{2}=E_{1/2}-\frac{RT}{2F}\ln\;K.$$

For the difference of formal potentials is valid

(11) $$E_{1}-E_{2}=\frac{RT}{F}\ln K.$$

If *K* = 1, all tree potentials are identical (*E*_1_ = *E*_2_ = *E*_1/2_). If *K* > 1, potentials decrease in the direction *E*_1_ > *E*_1/2_ > *E*_2_. The value of the constant *K* was calculated from potentials read in 1/4, 1/2, and 3/4 of the DC-tast wave using [18]:

(12) $$\begin{array}{cc} & K=\frac{{\left(10^{\left(E_{i}\cdot 2F)/(2\cdot 3RT\right)}\;-\;3\right)\;}^{2}}{10^{{\;}^{\left(E_{\text{i}}\cdot 2F)/(2\cdot 3RT\right)}}},\;\;\;\;\;\;\\ & \text{where}\;E_{i}\;=\;\left|\frac{E_{1}}{4}-\frac{E_{1}}{2}\right|\;=\;\left|\frac{E_{1}}{2}-\frac{E_{3}}{4}\right|. \end{array}\;$$

  The shape of the corresponding voltammetric curve is influenced by the value of the semiquinone formation constant (from 0 to ∞). In alkaline media, *K* values of the studied quinoxalinones are <16 (e.g., for **I,** pH 9.4, *K* = 8.42), and the curve is flatter. Individual reduction steps are not separated from each other. As acidity increases, *K* values grow significantly (e.g., for **I, **pH 2.9, *K* = 1262). As a result, the original two-electron single-wave signal splits into two individual waves. The effect becomes even more obvious with increasing *K* values. For compounds of types** I** and **II** this phenomenon can be observed in solutions of pH < 7.5 only. 

**Figure 8:**
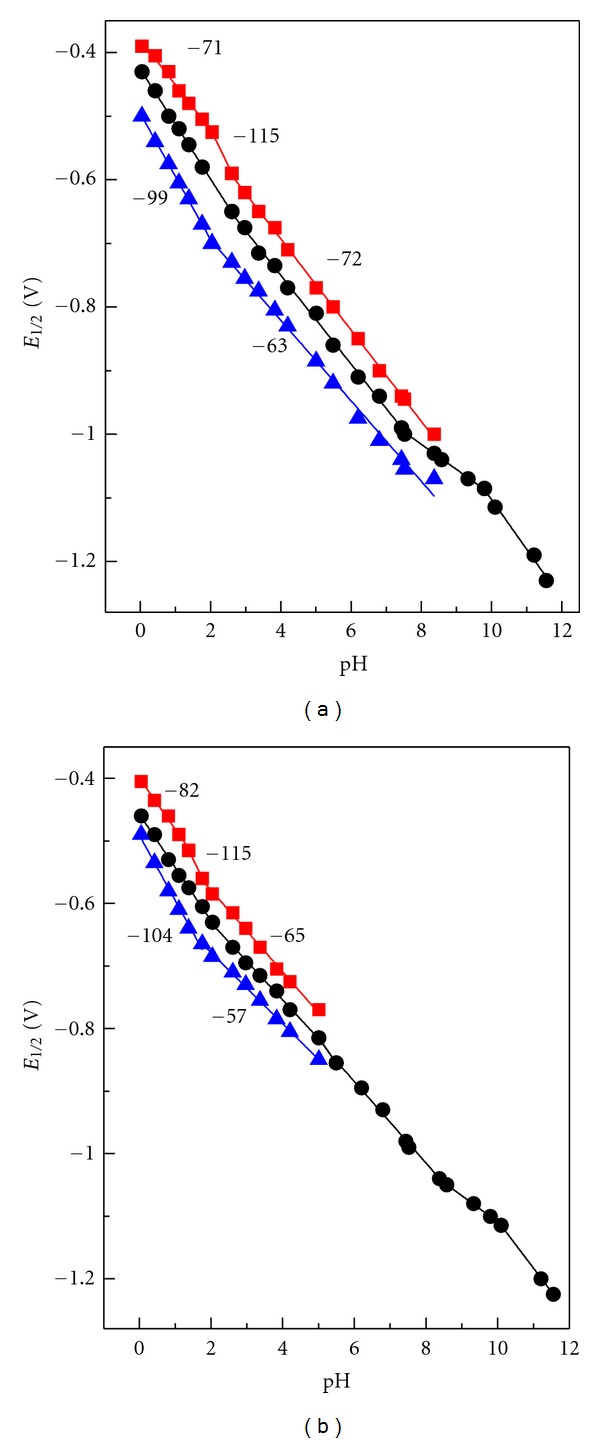
Dependencies of half-wave potentials of consecutive (red and blue symbols) and overall (black symbol) DCwaves for **I** (a) and **III** (b) on pH in HCl and Britton-Robinson solutions. The numbers indicate slopes (mV/pH) of particular linear segments for consecutive processes.

**Figure 9:**
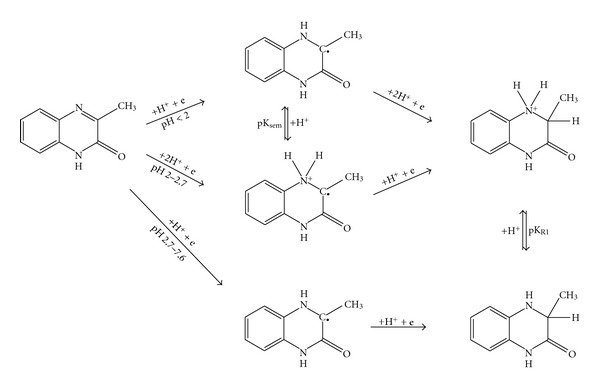
Detailed reduction scheme for **I** involving the semiquinone radical formation.

As for semiquinone formation, an initial electron incorporation into the position 4 is supposed by Pflegel and coauthors [9]. However, such a mechanism seems unlikely with respect to distribution of the electron density in the molecule. Moreover, a positive inductive effect of methyl group in position 3 together with the increased basicity of the N4-atom indicates that in a strongly acidic solution (pH < 2), protonation of the N-atom occurs at first. The gap in position 3 is then filled with an electron, forming a semiquinone free radical. Its reduction proceeds further by a reception of another electron and two protons. At pH 2.0–2.7 (semiquinone form prevailing), the reception of the electron and two protons is accomplished already in the first step (Figure 8(a) and 9). Within the range 2.7–7.6 (*pK*_*R*1_<pH<*pK*_*R*2_), the reduction process involves the reception of one electron and one proton in both steps producing a semiquinone. Two separate waves are approaching each other as pH increases, merging gradually into a single wave. At higher reactant concentrations, a possible semiquinone dimerization must also be taken into account [9], making the whole mechanism even more complex. 

**Figure 10:**
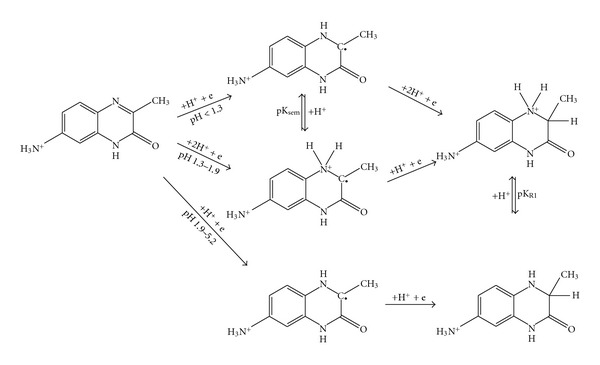
Detailed reduction scheme for **III** involving the semiquinone radical formation.

The reduction of the amino derivative **III** follows a similar pattern as that of** I **and** II **except for the semiquinone formation proceeding only in the pH range of prevailing protonized form, that is, pH < 5 (Figure 8(b)). At higher pH, the deprotonized amino group possessing a conjugated free electron pair accelerates the reduction process by its inductive effect to such an extent that the resulting semiquinones are not apparent in the form of separated peaks in the signal curve. Based on experimental data, a reduction scheme for **III **involving a formation of semiquinone can be deduced (Figure 10). 

#### 2.4.2. Adsorption

Adsorption is likely to affect the electrode processes by lowering the reaction reversibility. The effect was reported for** I** in pH range 0–4.5 as a prewave occurring in the more positive potential range characterizing adsorption of the reduced form [9]. 

**Figure 11:**
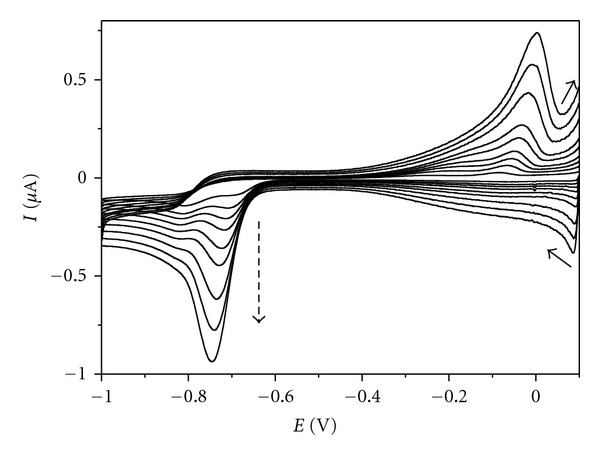
Cyclic voltammograms of **I** (*c* = 0.1 mM) in Britton-Robinson buffer (pH = 4) at different scan rates: 25, 50, 75, 100, 150, 200, 300, 400, and 500 mV/s. Dashed arrow indicates the direction of increasing scan rate.

Cyclic voltammograms (Figure 11) demonstrate the rather poor electrode process reversibility of quinoxalinones. The semiquinone formed during the first reduction can be reoxidized in the reversed scan. However, the difference in cathodic and anodic peak potentials is significantly higher than the theoretical value of 59 mV (as expected for reversible one-electron process), and the difference increases with the increasing scan rate (*v*). Dependencies log *I*_*p*_ = *f*(log⁡*v*) are linear within the whole pH range with slopes different for both the first and the second cathodic peak (Table 4).

The comparison of the line slopes with the analogous data for both purely diffuse (Randles-Sevcik equation) and tensametric currents indicates that adsorption occurs in acidic and neutral milieu, especially for** I **and** II **during the first reduction step (slopes 0.6–0.8) [14]. The adsorption of **III** in acidic medium is less evident. At neutral pH, with prevailing electroneutral oxidized form, the adsorption becomes more significant. 

During the second step of reduction and in alkaline solutions, the adsorption takes no effect for any of the analytes. Under such conditions, with the slope values smaller than 0.5 characterizing purely diffuse process, other factors (kinetic and catalytic) influencing the electrode processes cannot be excluded. 

The influence of adsorption on electrochemical reduction was confirmed also by elimination voltammetry with linear scan (EVLS)[19, 20]. The elimination function which eliminates simultaneously charged and kinetic currents and conserves diffusion current [19]:

(13) $$f\left(I\right)=17.485\;I-11.675\;I_{1/2}-5.8284\;I_{2}$$

was used. Symbol *I* denotes the reference voltammetric current measured at reference scan rate, *I*_1/2_ and *I*_2_ are currents measured at half and double scan rates, respectively. The theoretical elimination curve *f*(*I*) for an adsorbed electroactive substance has a characteristic peak-counterpeak form [21]. 

**Figure 12:**
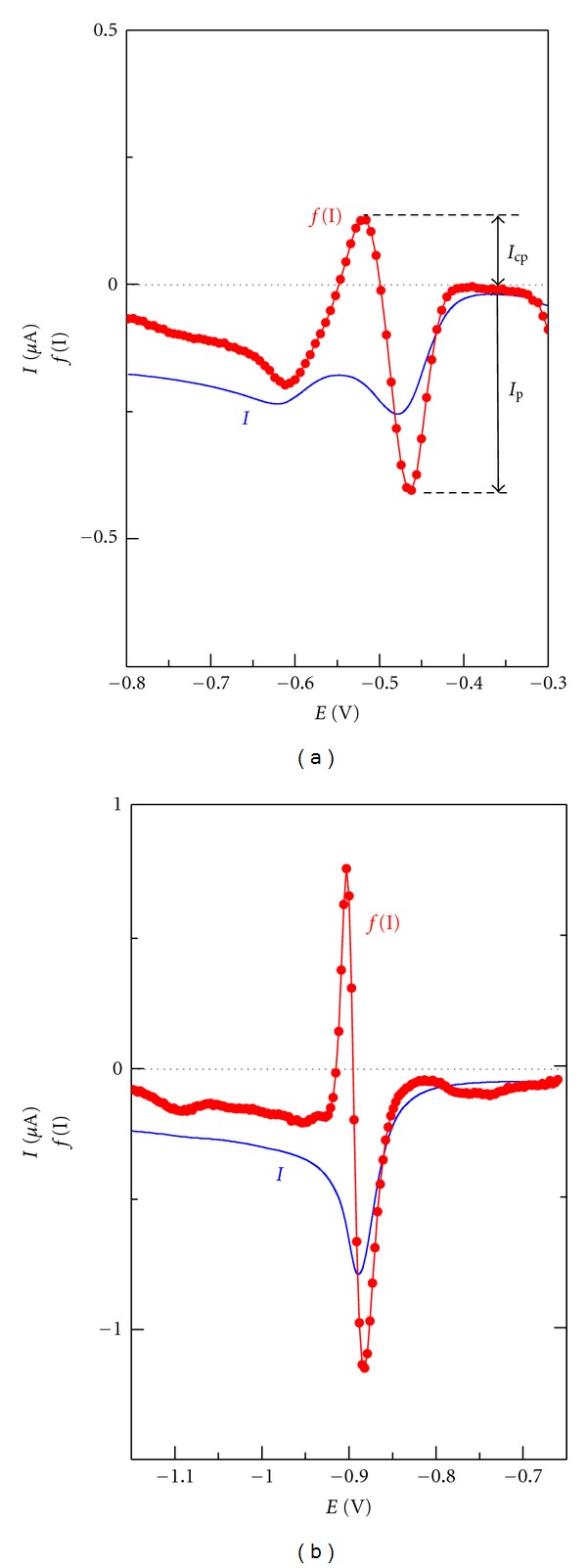
Linear sweep and elimination voltamograms of** I **in 0.01 M HCl (a) and **III** in phosphate buffer of pH 6 (b). LSV at reference scan rate 0.04 V s^1^. The elimination function *f*(*I*) eliminates simultaneously charging and kinetic currents and conserves diffusion current.

As can be seen from Figure 12(a), the elimination function *f*(*I*) calculated for the species I in 0.01 M HCl exhibits a peak-counterpeak shape at potential of the first cathodic peak. Similar shape of the elimination function was observed also with the substance **II **in acidic and neutral media confirming the influence of adsorption on the first step of electrode process. The theoretical ratio:

(14) Ip/(Ip  +  Icp),

where *I*_*p*_ and *I*_cp_ are the heights of peak and counterpeak of the elimination curve, respectively, has the value of 0.4097 for a totally adsorbed species reducing without a preceding chemical reaction [22]. In case of species **I** and **II** the ratio (14) was 0.76 and 0.55, respectively, in 0.01 M HCl at *I*_ref_  = 40 mV/s and increased with increasing pH of solution. This implies the growing influence of kinetics of the preceding chemical reaction (most likely protonation of the oxidized quinoxalinone form). In alkaline solutions where the anionic form of both substances prevails, the courses of the elimination function provided no evidence of adsorption. 

The derivative **III **revealed the elimination curve with peak-counterpeak shape in neutral solutions (Figure 12(b)) where the uncharged form of molecule prevails. The ratio (14) has value of 0.60 at pH 6 and *I*_ref_  = 40 mV/s. The elimination curves indicated no influence of adsorption for both the strong acidic media with prevailing protonized state of the **III **and the alkaline media with anionic form of quinoxalinone. In conclusion, the elimination voltammograms confirmed that the predominantly uncharged oxidized form of quinoxalinones adsorbs at the surface of mercury electrode. 

#### 2.4.3. Determination of Quinoxaline-2(1H)-One Derivatives 

**Figure 13:**
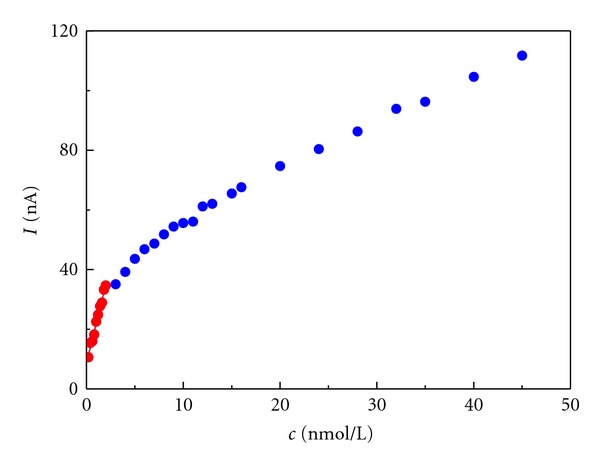
Concentration dependency of peak current for compound **I** in 0.1 M HCl measured by DPV.

Alkaline media (0.001 M NaOH) are generally suitable for determination of** I **and** II **derivatives by the DPV method. A single peak in DP-voltammograms at *E*_*p*_ = –1.042 V was obtained. Calibration curves were linear in the concentration range 10–0.5 *μ*M (Table 5). Concentration dependencies exhibit nonlinearity (Figure 13) in acidic and neutral media due to adsorption complicating the reaction mechanism. However, linear dependency for** I **and **II **in 0.1 M-HCl for low concentrations was obtained (*c* < 2 *μ*M). 

Neutral solution has proven to be suitable for the amino derivative **III**, for which linear calibration curves in the concentration range 0.1–1.0 *μ*M using DPV and DPP were obtained. Calibration curves become nonlinear at higher concentrations. Main parameters of the calibration curves and the reached limits of detection (LODs) are summarized in Table 5. 

Determination of low concentrations of **I–III** in model samples was performed at the same conditions as the corresponding calibrations. The resulting data are summarized in Table 6. 

Because of their biologically activity, the need for analytical determination of quinoxalinones in body fluids can be expected in the future. As a model experiment of their isolation from aqueous media for subsequent voltammetric determination, a solid phase as well as liquid-liquid extractions were performed. 

The solid phase extraction (SPE) was carried out using C18 SPE-cartridges containing 200 or 500 mg of sorbent. The cartridges were preconditioned using methanol and redistilled water. Samples of water (100 mL) spiked with 8–20 *μ*g of analyte were adjusted using buffer to final *pH* = 7.0 and supplied onto SPE cartridges through Teflon tubings (3–5 mL/min). Analyte elution from the drained SPE-cartridge was accomplished with 2 mL of methanol. The eluate was then dried by nitrogen stream at ambient temperature, redissolved in 1 mL of methanol, diluted to 10 mL with the background electrolyte (0.001 M-NaOH for** I **and **II**, neutral buffer for **III**) and subjected to DP-voltammetric analysis using the standard addition method. Recovery ranged between 56 – 62% for **II** and and 44 – 50% for **III **indicating the C18 stationary phase is not very suitable for the preconcentration of these compounds from water samples. Due to the low recovery, the described SPE procedure cannot be recommended for analytical purposes. 

Seven various solvents were tested for liquid-liquid extraction: hexane, benzene, dichloromethane, trichloromethane, carbon tetrachloride, diethyl ether, and ethyl acetate. For separation procedure, see Experimental. Ethyl acetate-water sample (2 : 1 by volume) and extraction time 5 min proved to be the most suitable preconcentration procedure. Distribution ratio for the species **I** was 8.5, extraction recovery achieved 94.5% at 20°C. 

## 3. Conclusions 

The study of electrochemical behavior of three quinoxaline-2-one derivatives at mercury dropping electrode has proven the pyrazine ring to be the electroactive center undergoing a two-electron reduction. The electrode process is pH-dependent. The protonization of nitrogen in position 4 precedes the electron acceptance forming a semiquinone radical which is relatively stable in acidic solutions. Its further reduction is manifested by separated current signals. A positive mesomeric effect of the nonprotonized amino group in position 7 of the derivative **III** accelerates the semiquinone reduction yielding a single peak. The final products of the electrochemical reduction are the corresponding 3,4-dihydroquinoxalin-2(1*H*)-ones. The electrode process is affected by the adsorption of the depolarizers on the mercury surface and is most pronounced when the quinoxalinone molecule is uncharged. The adsorption causes decreased reversibility of the overall electrode reaction and nonlinearity of concentration dependences. The suggested reaction mechanism was verified by the means of DC-polarography, coulometry with subsequent GC/MS analysis, and EVLS. The understanding of the mechanism was applied in developing of analytical method for the determination of the studied compounds. 

## Figures and Tables

**Table 1:** Comparison of spectrophotometrically and polarographically determined dissociation constants (derived from dependencies *E*_1/2_ = *f*(pH) at ionic strength of 0.15).

Compound	Polarography					Spectrophotometry
	*pK*_Ox1_	*pK*_Ox2_	*pK*_*R*1_	*pK*_*R*2_	*pK*_*R*3_	*pK*_*Ox*1_
**I**	9.9	—	2.7	7.6	—	9.85 (*λ* 286 nm)
**II**	9.8	—	2.0	8.0	—	10.04 (*λ* 300 nm)
**III**	5.2	10.1	1.9	~5.5	8.5	—

**Table 2:** DC-tast limiting current values of compared depolarizers (concentration 0.1 mM).

Compound	/[nA]		
	pH *≈* 1	pH = 7.0	pH = 9.2
*m*-nitrobenzoic acid	−689.0	—	−650.8
benzil	−344.7	−330.1	—
3-methyl-quinoxalin-2(1*H*)-one	−344.1	−336.8	−338.6

**Table 3:** Values of product **α*z* and number of protons *p* calculated from DC curves for compounds I, II, and III.

Compound	pH	**α*z *	**α*z *	Mean value		*p*	Slope calculated [V/pH]	Slope read off from the graph [V/pH]
		(a)	(b)	**α*z*	**α**			
**I**	<2.7	0.365	0.373	0.369	0.185	0.532	−0.088	−0.088
	2.7–7.6	0.451	0.482	0.467	0.223	0.553	−0.070	−0.070
	7.6–9.9	1.221	1.296	1.259	0.629	0.797	−0.038	−0.039
	>9.9	0.720	0.720	0.720	0.360	0.955	−0.079	−0.078

**II**	<2.0	0.329	0.281	0.305	0.153	0.398	−0.077	−0.077
	2.0–8.0	0.335	0.313	0.324	0.162	0.408	−0.074	−0.073
	8.0–9.8	0.739	0.810	0.774	0.387	0.595	−0.037	−0.036
	>9.8	1.136	1.118	1.127	0.564	1.166	−0.063	−0.063

**III**	<1.9	0.368	0.397	0.382	0.191	0.583	−0.091	−0.086
	1.9–5.2	0.755	0.664	0.698	0.349	0.689	−0.058	−0.062
	5.2–8.5	1.539	1.485	1.512	0.756	1.734	−0.068	−0.065
	8.5–10.1	1.385	1.316	1.351	0.676	0.931	−0.041	−0.045
	>10.1	0.649	0.651	0.650	0.325	0.856	−0.061	−0.076

**Table 4:** Slopes of the regression lines calculated for log-log dependencies of cathodic CV-peak current [nA] on scan rate [mV/s].

Medium	3-methyl-quinoxalin-2-one		3,6,7-trimethyl-quinoxalin-2-one		7-amino-3-methyl-quinoxalin-2-one	
	1st peak	2nd peak	1st peak	2nd peak	1st peak	2nd peak
0.01 M HCl	0.76	0.33	0.76	0.33	0.59	0.33
Phosphate pH 7.0	0.73	0.35	0.84	0.29	0.68	—
0.001 M NaOH	0.46	—	0.38	—	0.41	—

**Table 5:** Regression parameters of calibration curves *y = ax + b *(significance level **α**= 0.05, number of calibration points *n* = 10).

Analyte	Method (*E *_P_ [V])	*c *[*μ*M]	Electrolyte	*a* ± *s*_*a*_ [nA/*μ*M]	*b* ± *s*_*b*_ [nA] [nA]	*r*	LOD [*μ*M]
I	DPV (−1.075)	0.3–10.0	0.001 MNaOH	2.74 ± 0.03	0.41 ± 0.21	0.9994	0.23
I	DPV (−0.457)	0.4–2.0	0.1 M HCl	12.39 ± 0.23	−0.01 ± 0.30	0.9988	0.07
II	DPV (−1.010)	0.3–10.0	0.001 M NaOH	2.73 ± 0.03	0.25 ± 0.19	0.9995	0.21
III	DPV (−1.100)	0.1–1.0	B.-R.-buffer pH 7.0	26.85 ± 0.21	−0.17 ± 0.13	0.9998	0.01
III	DPP (−1.080)	0.1–1.0	B.-R.-buffer pH 7.0	23.69 ± 0.13	−0.18 ± 0.08	0.9999	0.01

*E_P_* versus SCE,* r*—correlation coefficient, LOD = 3*s*_*b*_/*a.*

**Table 6:** Determination of derivatives of I–III, method of standard additions (number of measurements *n* = 4).

Analyte	Method	Mean content $\overset{®}{x}\;$[*μ*g/mL]	RSD *s*_*r*_[%]	Relative mean error **ε** [%]	Electrolyte
I	DPV	0.160	3.23	−0.24	0.001 M NaOH
I	DPV	0.065	3.02	3.08	0.1 M HCl
II	DPV	0.192	3.68	1.85	0.001 *M* NaOH
III	DPV	0.0187	1.84	4.84	B.-R. buffer, pH 7.0
III	DPP	0.0170	2.10	−3.25	B.-R. buffer, pH 7.0
